# Study on vector mosquito of zoonotic *Brugia malayi* in Musi Rawas, South Sumatera, Indonesia

**DOI:** 10.14202/vetworld.2019.1729-1734

**Published:** 2019-11-07

**Authors:** Budi Mulyaningsih, Sitti Rahmah Umniyati, Suwarno Hadisusanto, Erwin Edyansyah

**Affiliations:** 1 Department of Parsitology, Faculty of Medicine Public Health and Nursing, Universitas Gadjah Mada, Yogyakarta, Indonesia; 2 Department of Tropical Biology, Faculty of Biology, Universitas Gadjah Mada, Yogyakarta, Indonesia; 3 Postgraduate Program of Medicine, Faculty of Medicine Public Health and Nursing, Universitas Gadjah Mada, Yogyakarta, Indonesia

**Keywords:** *Armigeres subalbatus*, *Brugia malayi*, lymphatic filariasis, Musi Rawas, South Sumatera

## Abstract

**Background and Aim:**

Studies to determine abundance, distribution, species composition, and mosquito interactions are very important in understanding the risk of disease transmission to implement appropriate mosquito management in endemic areas. Lymphatic filarial worms are one of the parasites that are contracted and/or transmitted by mosquitoes when sucking the blood of infected humans or animals and then biting others. This research was conducted to study the abundance, species composition, mosquito biting cycles, density and periodicity of mosquitoes caught in Lubuk Pauh Village, Bulang Tengah Suku Ulu, Musi Rawas, South Sumatera, Indonesia, which is an endemic area of zoonotic *Brugia malayi*.

**Materials and Methods:**

The mosquito collection was done in July 2018 using the human landing collection method for 11 h from 18.00 pm to 5.00 am Western Indonesian Time. The catching of mosquitoes was done both indoors and outdoors, and mosquitoes were identified under a dissecting microscope using an identification key to confirm their species. Detection of *B. malayi* larvae in mosquitoes was confirmed by dissection and polymerase chain reaction methods.

**Results:**

The caught mosquitoes consisted of four species: *Armigeres subalbatus*, *Culex quinquefasciatus*, *Culex vishnui*, and *Mansonia uniformis*. Based on the Shannon–Wiener index, Lubuk Pauh Village has low mosquito species diversity (0.210). *Ar. subalbatu*s was the dominant mosquito in Lubuk Pauh Village with dominance number 95.08, and it had the most frequent activity in each of periods of indoor and outdoor collection, with the highest density (man-hour density) at 18.00-19.00 (51.750). *B. malayi* infective stage larvae were not found in all mosquito species caught.

**Conclusion:**

Existence of *Ar. subalbatus*, *Cx. quinquefasciatus*, and *Ma. uniformis* in Lubuk Pauh Village which is an endemic area of *B. malayi* shows that the area is at risk of lymphatic filariasis transmission.

## Introduction

Lymphatic filariasis, commonly called elephantiasis, is one of the neglected tropical diseases. At present, 856 million people in 52 countries are living in areas that require preventive chemotherapy to stop the spread of this infection [1]. Epidemiologically, Indonesia is in an area that is at high risk of contracting filariasis [2]. In South Sumatera Province, lymphatic filariasis is present in almost all districts. Musi Rawas Regency is one of the endemic areas of filariasis and in 2014-2016, 29 chronic sufferers were reported in three villages [3]. Lymphatic filarial worms are one of the parasites that are contracted and/or transmitted by mosquitoes when sucking the blood of infected humans or animals and then biting others. The ability of vector mosquitoes to swallow filarial worms and to support their development after being in their bodies is an important determinant of transmission of filariasis. The amount of microfilaria that is sucked and that develop into infective stage larvae (L3) is not constant and depends on many factors that influence their maturity. Lymphatic filariasis is transmitted by many species of mosquitoes in four principal genera—Anopheles, Culex, Aedes and Mansonia, the distribution, ecology, biology and transmission potential of which vary greatly. As transmission efficiency differs considerably by vector species, it is important to understand the entomological aspects of transmission of lymphatic filariasis. Mansonia spp is the main vector of zoonosis malayan filariasis [4]. Lymphatic filariasis is caused by three filarial worm species, namely, *Wuchereria bancrofti*, *Brugia malayi*, and *Brugia timori*. All of these species are found in Indonesia, but more than 70% of filariasis cases in Indonesia are caused by *B. malayi*. Malayan filariasis is one of the zoonotic diseases that can be transmitted from animals to humans. This disease has reservoir hosts and mosquito vectors. Humans are considered the main definitive host of filariasis, but there are several types of animals that can act as sources of filariasis transmission. Of all the filarial worm species that infect humans in Indonesia, only *B. malayi* nocturnal subperiodic type, and non-periodic type are found in animals, namely, monkey (*Macaca fascicularis*), lutong or black monkey (*Presbytis cristatus*), and cats (*Felis catus*). The presence of animals that become filariasis reservoir host will be one of the problems in efforts to eliminate filariasis in Indonesia [5]. Research in Narathiwat Province, Southern Thailand, from the results of 2515 cats, 401 cats were positive for microfilaria *B. malayi* [6] and in East Kalimantan from the results of microfilariae examination in 645 cats found 14 cats positive for microfilaria *B. malayi* [7].

At present, there are 23 species of mosquitoes from the genera *Anopheles*, *Culex*, *Mansonia*, and *Armigeres* that can act as filariasis vectors in Indonesia [2]. The study aimed to determine density, abundance, distribution, and species composition, and host and mosquito interactions are very important in understanding the risk of disease transmission to apply appropriate mosquito management. Thus, understanding the factors that modulate vectors and host distribution, density, and abundance, as well as vector mosquito biting behavior, are important steps in characterizing the risk of transmission and consequences of vector-borne diseases.

## Materials and Methods

### Ethical approval

This research was conducted in July 2018 and has been approved by the Medical and Health Research Ethics Commission of the Faculty of Medicine, Public Health, and Nursing Universitas Gadjah Mada, with numbers KE/FK/0389/EC/2018. The design of this study was cross-sectional with a spot survey design.

### Study sites

The study was conducted at four hamlets (Hamlet I, II, III, and IV) in Lubuk Pauh Village, Bulang Tengah Suku (BTS) Ulu Sub-district, Musi Rawas District, South Sumatera Province, Indonesia. Musi Rawas district is one of the endemic areas of Malayan filariasis in South Sumatera.

### Mosquito sampling

A mosquito sampling was done using human landing collection (HLC) in two houses in every hamlet in Lubuk Pauh Village for 11 h from 18.00 pm to 05.00 am Western Indonesian Time (WIT). Catching mosquitoes were done both indoors and outdoors by a team of four trained volunteers per house, with two volunteers sitting in the house, and two volunteers sitting outside the house. To determine mosquito biting cycles, HLC was conducted hourly for 40 min for the two groups (indoors and outdoors), each with two persons, for two consecutive nights. Mosquitoes caught were put in paper cups covered with net and then sorted and identified morphologically using the identification keys with a dissecting microscope.

### Detection of microfilaria/larva B. malayi in mosquitoes

Each species of mosquito caught, which was considered to be able to act as a vector of *B. malayi* was examined by dissection and molecularly with polymerase chain reaction (PCR) methods to determine whether there were microfilariae or *B. malayi* larvae by pooling method (10-25 mosquitoes). In this method, a group of mosquitoes was put on the object-glass, some drops of physiological saline solution were added, and the mosquitoes were pressed with another object-glass, until the body of the mosquitoes separated into several parts. Mosquitoes were then moved into a Petri dish which has been filled with physiological saline solution to soak the body parts of the mosquitoes. The immersion of the mosquitoes was left for 5-10 min, and the Petri dish was observed under the dissecting microscope.

The procedure for the detection of microfilariae or larvae of *B. malayi* in mosquitoes by pooling method of 10-25 mosquitoes using PCR method was carried out through several activities as following: DNA isolation, running PCR, and electrophoresis. Female mosquitoes were collected based on species. Each pool then homogenized with a pestle in a tube microcentrifuge containing 180 µl phosphate buffer saline (pH 7.2). DNA was further extracted from each tube using Genomic DNA mini Kit GeneaidTM Cat No. GB100. Lot No.JM02202, according to company protocol. Amplification of PCR is carried out with thermocycler using HhaI forward primers (5 ‘GCGCATAAATTCATCAGC-3’) and reverse HhaII (5’GCGCAAAACTTAATTACAAAAGC-3’). The PCR mixtures contained 15 μl of Mix PCR (GoTaq^®^ Green Master Mix. 2×), 11 μl of ddH2O (nuclease-free water lot. 0000123190 Promega), 2 μl of R and F primers (20 μM), and 2 μl of the DNA template in a total volume of 30 μl. The temperature was programmed under the following conditions: One cycle of initial denaturation at 94°C for 5 min, followed by 40 cycles of 94°C for 1 min (denaturation), 56°C for 1 min (annealing), and 72°C for 1 min (extension); and a final extension step at 72°C for 10 min. Following the PCR 20 μl of each PCR product was electrophoresed on a 2% agarose gel, stained with ethidium bromide, and observed under UV. Mosquito samples were found positive for the filarial parasite (*B. malayi*), if exhibited amplification of 322 bp DNA fragment [8].

### Statistical analysis

The diversity of mosquito species was analyzed by Shannon–Wiener diversity index [9]. Other entomological indexes were calculated, such as relative abundance, species frequency, the dominant number, periodicity of mosquito, and species mosquito density [10].

## Results

### Species diversity-dominance, relative abundance frequency of mosquitoes

During the collection period with the HLC for 11 h (18.00-05.00 WIT) in Lubuk Pauh, at coordinates S 3° 13’ 17.3’’; as many as, 630 mosquitoes were obtained consisting of four mosquito species. Based on the Shannon–Wiener index, Lubuk Pauh Village has low mosquito species diversity (0.210). Diversity of species was expressed by H index (H1), which included the low category if the number is <1, medium category if the number is more than 1, and <3 and high category if the number is more than 3 [11]. Species diversity of mosquitoes caught in Lubuk Pauh Village, as shown in Table-1.

**Table-1:** Species diversity of mosquitoes caught in Lubuk Pauh village by human landing collection method.

Mosquito species	Human landing collection		Total

	Indoors	Outdoors	
*Armigeres subalbatus*	206	393	599
*Culex quinquefasciatus*	11	18	29
*Culex vishnui*	1	-	1
*Mansonia uniformis*	1	-	1
Number of mosquitoes	219	411	630

Based on the composition of the species of mosquito caught, *Armigeres subalbatus* was the dominant mosquito species (599). This study also showed that the number of mosquitoes collected outdoors (411) was higher than those caught indoors (219). Mosquitoes collection were done for two consecutive nights and there were 22 collecting hours. The relative abundance, species frequency, and dominant species of mosquito species caught in Lubuk Pauh Village with HLC method are shown in Table-2.

**Table-2:** The relative abundance, species frequency, and dominance numbers of mosquitoes caught in Lubuk Pauh Village with the human landing collection method.

Mosquito species	Number of mosquitoes	Human landing collection		

		Relative abundance (%)	Species frequency	Dominance numbers
*Armigeres subalbatus*	599	95.08	1.00	95.08
*Culex quinquefasciatus*	29	4.60	0.86	3.96
*Culex vishnui*	1	0.16	0.04	0.006
*Mansonia uniformis*	1	0.16	0.04	0.006

Species frequency without %, Dominance numbers without %

### Density and periodicity of *Ar. subalbatus*

*Ar. subalbatus* was the dominant mosquito in Lubuk Pauh Village, and it had the most frequent activity in each of the periods, both indoor and outdoor collection. Density (man-hour density) of *Ar. subalbatus* is presented in Table-3. Table-3 shows that *Ar. subalbatus* is active all night both indoors and outdoors, with the highest density (man-hour density) at 18.00-19.00 WIT, which is equal to 51.750.

**Table-3:** The MHD of *Ar. subalbatus* from Lubuk Pauh village, July 2018.

Mosquito collecting time	*Armigeres subalbatus* density (MHD)		Total of MHD

	Indoors	Outdoors	
18.00-19.00	17.250	34.500	51.750
19.00-20.00	7.500	16.500	24.000
20.00-21.00	5.625	11.625	17.250
21.00-22.00	7.125	9.000	16.125
22.00-23.00	7.500	9.000	16.500
23.00-24.00	7.125	10.500	17.625
24.00-01.00	4.875	13.875	18.750
01.00-02.00	5.625	11.250	16.875
02.00-03.00	6.000	7.875	13.875
03.00-04.00	5.625	10.875	16.500
04.00-05.00	5.250	9.000	14.250

MHD=Man-hour density

### Behavior of *Ar. subalbatus* in Lubuk Pauh village

**Figure-1:**
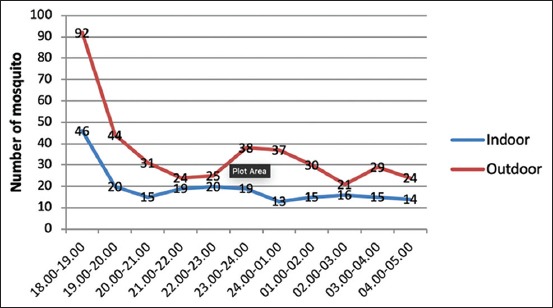
Periodicity of *Armigeres subalbatus* from Lubuk Pauh village.

All mosquito species have their own distribution, behavior pattern, and characteristics of its habitat, which are different from others. The daily behavior pattern of mosquito activities will occur at day or night time depending on the species. From the results of this study, it can be seen that the *Ar. subalbatus* is active all night from 18:00 to 05:00 WIT. The highest activity of *Ar. subalbatus* that was collected on human bait was between 18:00 and 19:00 WIT both indoors and outdoors (Figure-1).

### *B. malayi* larvae detection in mosquitoes

Dissection of *Ar. subalbatus, Culex quinquefasciatus, Culex vishnui*, and *Mansonia uniformis* that were caught in Lubuk Pauh did not find filariasis larvae. Similarly, the molecular examination using the PCR methods also showed negative results. This result means that filarial larvae were not found in all mosquito species caught.

## Discussion

Lubuk Pauh Village is one of the endemic areas of Malayan filariasis in South Sumatera, Indonesia (S 3° 13’ 17.3’’). This village is still categorized as an underdeveloped village, located on the banks of the Musi River with a typology of rubber plantation land so that the majority of the population works as rubber farmers. In this area, four species of mosquitoes were found, which included *Ar. subalbatus, Cx. quinquefasciatus, Cx. Vishnui*, and *Ma. uniformis*, with low mosquito species diversity (Shannon–Wiener index 0.210). This finding is probably due to the landing collection of the mosquitoes that were only done twice in July 2018 because it was a preliminary study to find out the picture of mosquitoes found in endemic areas of Malayan filariasis. At that time, in the area, it was in the dry season and experiencing drought. Mosquitoes are cosmopolite insects, which are widespread in the tropic and sub-tropic regions. Seasonally, the changing of the environment also affects their activity and impacts on their diversity, distribution, and density. Research has shown that mosquitoes can be competent vectors, efficient distributors, and reinforcing agents for various parasites in animal and human populations [11,12].

The most dominant mosquito caught was *Ar. subalbatus*, which is commonly found close to human dwellings, especially in suburban areas with poor sanitation that contains polluted water such as septic tanks. The density (man-hour density) of *Ar. subalbatus* outdoors was higher than indoors. This was allegedly due to the character of *Ar. subalbatus* that is more exophilic. *Ar. subalbatus* is closely linked with artificial habitats and also breeds in tree holes and has been known to be a vector of Japanese encephalitis virus, a vector of filarial worm *W. bancrofti* in India and the dog heartworm *Dirofilaria immitis* in Peninsular Malaysia [13-15]. This mosquito species is also the vector for the zoonotic *Brugia pahangi* infection in Malaysia, and therefore should now be categorized as a medically important mosquito species. Its central role in the transmission of zoonotic *B. pahangi* must be considered important in future studies on filarial infections [16].

In this study, the dominance numbers *of Cx. quinquefasciatus, Cx. vishnui*, and *Ma. uniformis* were very low. *Cx. quinquefasciatus* is a vector of the *W. bancrofti* urban type, and in Pekalongan (Central Java), Indonesia, it has been confirmed that *Cx. quinquefasciatus* is a *W. bancrofti v*ector. Shriram *et al*. reported that *Cx. quinquefasciatus* is a *W. bancrofti* diurnal subperiodic type vector in Nicobarase, Nicobar Island, and India [17]. Iris *et al*. also reported that *Cx. quinquefasciatus* is a *W. bancrofti* vector in Tanzania [18]. In the research conducted by Safitri on *Cx. quinquefasciatus* from Barito Kuala District, Kalimantan, *B. malayi* larvae were found [19]. This finding can happen because the environment in these places is suitable for *Cx. quinquefasciatus* and *B. malayi* worms. Research conducted by Yahya *et al*. shows that *Cx. quinquefasciatus* has more potential to be a *B. malayi* vector than *Ar. subalatus* [20].

Very few *Ma. uniformis* were caught in Lubuk Pauh Village, BTS Ulu Sub-district, Musi Rawas District, South Sumatera Province, Indonesia. The previous study showed that *Ma. uniformis* in Sedang, Suak Tapeh, Banyuasin, South Sumatera, and also in Karanganyar Village, Banyuasin, South Sumatera were the dominant mosquito [21,22]. The Indonesian Ministry of Health reported that in South Sumatera, filariasis is caused by *B. malayi* and the vector is *Ma. uniformis* [23]. The character of *Ma. uniformis* breeding places can affect the incidence of lymphatic filariasis. The research of Sapada *et al*. in Banyuasin (South Sumatera) and Zen in East Lampung showed that environmental conditions with many aquatic plants such as swamps and ponds can be ideal breeding places for *Mansonia* spp. and are associated with the incidence of lymphatic filariasis [24,25]. *Ma. uniformis* is the main vectors of zoonotic *B. malayi* nocturnal subperiodic type in South Sumatera Province, Indonesia, and in Southeast Asia, however, in Africa, *Ma. uniformis* and *Mansonia africana* are the main vectors of *W. bancrofti* [5,23,26]. Studies of bloodsucking mosquito activity can be used to take preventive measures to avoid filariasis vector mosquito bites. Many factors can influence potential vectors to be positive for microfilaria including the amount of microfilariae that are sucked is sufficient or not to develop in the body of a mosquito. The requirements for mosquitoes to become vectors include the age of mosquitoes, contact between humans and animals (hosts) with mosquitoes, the frequency of bloodsucking, and the susceptibility of mosquitoes to parasites [10]. The estimated capacity to be a vector is influenced by environmental, behavioral, biochemical, and cellular factors that influence the relationship between vectors, pathogens that will be transmitted by vectors, and hosts where the pathogen will be transmitted. Behavior and environmental factors have a role in distinguishing the mosquitoes’ capacity as vectors [27]. Knowledge of mosquito endemicity and mosquito vector density is very important as important parameters to assess the success of vector control programs [2].

In this study, no filariasis larvae were found in all mosquitoes caught by dissection and PCR methods, so the infection rates could not be determined. Thus, filariasis vectors in this area cannot be determined because the discovery of filariasis larvae in the mosquito’s body is needed to confirm mosquitoes as vectors. In addition, the discovery of *Ar. subalbatus*, which is very dominant in Lubuk Pauh Village, can also be considered as a filariasis vector because in Papua *Armigeres* spp. have also been identified as filariasis vectors makers of human and animal-sourced disease control [23]. The existence of certain mosquito species, especially mosquitoes transmitting diseases in an area can be important information to be followed up. The results of this study can be used as supporting data for programs in conducting vector control activities. Continuous (longitudinal) observations of certain species as vectors need to be done routinely to provide more complete and useful information for policy programs.

## Conclusion

In Lubuk Pauh Village as a nocturnal subperiodic *B. malayi* endemic area, four mosquito species were caught, and *Ar. subalbatus* was the dominant species, and it had the most frequent activity in each of the periods, both indoor and outdoor collection. Other mosquitoes collected were *Cx. quinquefasciatus, Cx. vishnui*, and *Ma. uniformis* although in small numbers. *B. malayi* larvae were not found in all mosquito species caught; however, the discovery of *Ar. subalbatus*, *Cx. quinquefasciatus*, and *Ma. uniformis* indicates that the area is at risk of transmission of lymphatic filariasis. Further studies need to be carried out with several mosquito samples to illustrate the effect of climate on the presence of mosquitoes in an area.

## Authors’ Contributions

BM designed the study, SRU and SH conducted the field survey, and EE collected mosquito samples. All authors drafted, revised, read, and approved the final manuscript.
